# Effect of Instant Messaging-Based Integrated Healthcare on Medical Service Use and Care Outcomes in Patients with Disabilities

**DOI:** 10.3390/healthcare13111335

**Published:** 2025-06-03

**Authors:** Han-Chin Hsieh, Yan-Yuh Lee, Nai-Ching Chen, Ya-Chuan Hu, Lin-Yi Wang

**Affiliations:** 1 Department of Physical Medicine and Rehabilitation, Kaohsiung Chang Gung Memorial Hospital, Chang Gung University College of Medicine, Kaohsiung 83301, Taiwan; kghschin@cgmh.org.tw; 2 Riverbank Rehabilitation Clinics, Kaohsiung 83301, Taiwan; newstartqq@gmail.com; 3 Department of Neurology, Kaohsiung Chang Gung Memorial Hospital, Chang Gung University College of Medicine, Kaohsiung 83301, Taiwan; naiging@yahoo.com.tw; 4 Administrative Department, Kaohsiung Chang Gung Memorial Hospital, Chang Gung University College of Medicine, Kaohsiung 83301, Taiwan; huya0429@cgmh.org.tw

**Keywords:** integrated healthcare services, instant messaging, disability, polypharmacy

## Abstract

**Objectives:** We aimed to investigate how receiving integrated healthcare services from a case manager via instant messaging affected patients with disabilities. **Methods**: This database-matched case–control study was conducted at one medical center. Patients with officially certified disabilities were recruited and assigned to either the LINE-based group or the control group, which accessed services in the traditional manner. Their baseline characteristics were collected through chart reviews. Medical service utilization data—including their number of outpatient visits, prescribed medications, and hospitalizations—were obtained at baseline and 3, 6, and 12 months into the intervention. In the LINE group, quality of life, caregiver burden, and perceived social support were also assessed. A repeated-measures ANOVA was used to analyze within- and between-group differences over time. **Results**: Both the LINE group and the control group contained 66 patients. The number of outpatient visits (*p* < 0.001) and quantity of medication taken (*p* = 0.026) were significantly lower in the LINE group than in the control group. Furthermore, the caregiver burden in the LINE group (*p* = 0.024) was significantly lower 12 months after receiving integrated healthcare services. **Conclusions**: Providing integrated healthcare services via instant messaging enabled patients with disabilities to access medical services promptly and efficiently, thus enhancing the accessibility of healthcare and improving care for the disabled population.

## 1. Introduction

Disability used to be defined as a limitation of an individual’s activity due to an underlying pathology or impairment. The World Health Organization’s (WHO) International Classification of Functioning, Disability, and Health defines disabilities as impairments, limitations in activity, and restrictions in participation, which better illustrate the negative impact of disability on an individual at the environmental level [1]. In Taiwan, individuals with significant impairment or a loss of body structure or function that affects their ability to perform activities and participate in social life may apply for a disability certificate and are eligible for certain social welfare benefits, such as National Health Insurance subsidies or discounts on public transportation. So far, more than 1,170,000 people in Taiwan have registered as disabled and been issued disability certifications, which is approximately 5% of the population and a number that has increased gradually in the last few years [2]. The three most common categories of impairment registration are related to mental function and the structure of the nervous system, neuromusculoskeletal and movement-related functions and structures, and sensory functions including the eye, ear, and related Structures [2]. More than 60% of individuals with disability certifications require regular outpatient care; moreover, the medical needs of this population are greater than those of individuals without disabilities [3]. Patients with disabilities are disadvantaged when it comes to accessing healthcare, from making an appointment with a physician and entering healthcare facilities to receiving medical care. However, due to the complexity and multisystemic nature of their underlying diseases, they may require follow-ups that involve multiple medical specialties [4,5,6]. McColl et al. (2010) found that the causes of unmet healthcare needs in people with disability include costs and individual patient or provider factors, such as needs, expectations, communication, and structural issues [7,8]. As a result, there is a need to improve the relationship between the healthcare system and patients with disabilities in order to create a better quality of healthcare.

To overcome the barrier between individuals with a registered disability and the healthcare system, an interdisciplinary team has been established [9] and several modifications to the healthcare system have been made, such as seeing patients in alternative places, reducing movement between different floors in the hospital, examining patients in their wheelchairs to reduce the number of transfers required, making home visits, and communicating by phone, email, or Skype [6,10]. The case managers of these interdisciplinary teams—who are typically registered nurses, social workers, or allied health professionals—serve as key liaisons between physicians and patients, ensuring coordinated, patient-centered care. Their responsibilities include receiving referrals from healthcare institutions or family members and providing consultations on disability-related issues such as education, employment, social welfare, and financial aid. They conduct comprehensive needs assessments and develop individualized service plans in collaboration with patients and families, coordinating interdisciplinary meetings for care planning and connecting patients with resources. Additionally, case managers organize caregiver support programs and disability-related educational or therapeutic activities while also addressing the complex needs of aging families with intellectually disabled members through tailored support and integrated care planning.

Pre-existing integrated disability medical services primarily focus on hospital orientation, providing aid in clinics, making special appointments at clinics, and coordinating members of different specialties. Prompt and efficient communication between the healthcare system and individuals with disabilities is important to further improve the accessibility and availability of healthcare. Instant messaging from a case manager could enable this. As of 2016, LINE was the most popular instant messaging application in Taiwan, having been installed on 90% of smartphones as of 2014 [11]. This messaging app not only has a text messaging feature but also enables real-time voice and video calls [12]. Photos, voice messages, videos, and emojis (stickers expressing emotions) can easily be sent in individual or group chats [13]. Because of the ready availability of LINE on smartphones in Taiwan, this free instant messaging application may help to lower the barrier between individuals with registered disabilities and the healthcare system via the provision of integrated healthcare services. A qualitative study in 2016 found that LINE can improve the quality of care and promote nurse–patient relationships for patients with chronic diseases [13]. However, the study only evaluated the use of the instant messaging application from the nurses’ perspective and did not gather quantitative data and feedback from patients and their families. Another study conducted in 2014 demonstrated that Short Message Service (SMS; text messages) provided timely information that guided and supported women who underwent abortion in making that medical decision [14]. The results showed that medical advice could be efficiently provided to patients via mobile devices. Gilmore et al. found that health interventions delivered via smartphones did not outperform conventional care in terms of decreasing postpartum weight retention. Therefore, investigating the effect of instant messaging applications on various groups of patients is necessary [15]. Furthermore, to the best of our knowledge, there has been no case–control study designed to assess effective accommodations for patients with disabilities in primary care.

Therefore, this study aimed to investigate whether incorporating instant messaging (LINE) services into healthcare services would provide better care and access to medical resources for patients with disabilities.

## 2. Materials and Methods

### 2.1. Study Design and Participants

This study was a database-matched case–control study. The inclusion criteria were that patients (1) be at least 20 years of age and (2) have a certificate of disability and have made a healthcare request at a medical center between May 2017 and December 2021. Individuals were excluded if they were unable to cooperate with our evaluation, were experiencing severe illness resulting in unstable vital signs, exhibited significant mental or psychiatric disabilities that impaired their ability to participate in interviews, or declined to provide informed consent. Patients were recruited from a disability clinic with integrated healthcare services at a medical center in Taiwan. Patients and their caregivers were assessed for their ability to communicate via the LINE application on their smartphones. Those who demonstrated proficiency and expressed a willingness to use LINE to access integrated healthcare services were assigned to the LINE group. Within this group, participants communicated with one case manager via LINE, who scheduled appointments, managed cancelations, messaged healthcare services, and ensured their access to a hospital. In consideration of medical ethics, we did not randomize the disability group because they are an inherently vulnerable group; instead, a 1:1 age- and sex-matched control group was randomly selected from the same database of disabled patients, with additional matching for education level in the medical center. Participants in the control group continued to access healthcare services through conventional methods, including telephone, email, and websites.

On the other hand, those in the LINE group accessed integrated healthcare services, which provide information about health education, social welfare, and mental support and assist in making appointments.

The sample size was determined based on the number of patients visiting the medical center during the 3-year period of a government-initiated program in the rehabilitation department, which aimed to improve access to medical services for individuals with disabilities. As this study was conducted without dedicated funding, the number of participants was determined by the number of eligible patients encountered during routine clinical practice while the program took place. This approach reflects real-world constraints while aligning with the exploratory nature of this study.

This study was approved by the Institutional Review Board of our institution (IRB No. 201700505B0).

### 2.2. Sources and Measurement of Data

We reviewed patients’ medical charts and extracted demographic characteristics including their age, sex, and disability type. In addition, we recorded the patients’ clinic visits, medication, and hospitalization records at baseline and 3, 6, and 12 months after joining the integrated healthcare service for patients with disabilities. For the LINE group, we adapted the Taiwanese version of the WHOQOL-BREF (The World Health Organization Quality of Life Brief Version) in order to evaluate patients’ quality of life and used the Care Burden Index (CBI) and social support rating scale (SSRS) to evaluate the burden on patients’ unpaid major caregiver at baseline and 3, 6, and 12 months after joining the integrated healthcare service for patients with disabilities. The questionnaires were completed during clinic visits or via Google Forms.

### 2.3. The World Health Organization Quality of Life Brief Version (WHOQOL-BREF)

The WHOQOL-BREF (Taiwanese version) was developed from the WHOQOL-BREF by adding two Taiwanese culture-specific questions with good reliability and validity [16,17]. The 28 questions in this version consisted of physical health, psychological factors, social relations, and environmental aspects and are split as follows. There are seven questions on physical health, six on psychological health, four on social relationships, and nine on environmental health [16]. Each individual item of the WHOQOL-BREF is scored on a five-point ordinal scale from 1 to 5. The scores are then transformed to a 0–100 scale, with higher scores indicating a better quality of life. The physical health section includes questions on pain, energy, sleep, mobility, daily activities, medical-related dependency, and work capacity. The psychological questions cover negative thoughts, positive feelings, self-esteem, memory and concentration, self-image, and religion and spirituality. The social relationship section contains questions about personal relationships, social support, respect or acceptance, and sex life. The environmental health section covers issues related to physical safety, home environment, financial resources, health and social services, opportunities to acquire new skills and knowledge, recreation, general living environment, transportation, and diet [16].

### 2.4. Caregiver Burden Inventory (CBI)

The Caregiver Burden Inventory questionnaire consists of 24 items that measure six aspects of the caregiver’s burden: time dependency, physical health, development, social relationships, and emotional health. The scores for each item are given on a five-point Likert scale that ranges from 0 (not at all disruptive) to 4 (very disruptive). The total score is the summation of the scores for all 24 items, and a total score above 36 indicates a risk of “burning out” [18].

### 2.5. Social Support Rating Scale (SSRS)

The SSRS was developed to measure the perceived helpfulness of assistance provided to individuals [19,20]. The SSRS consists of ten items that cover subjective support (four items), objective support (three items), and the utilization of social support (three items) [21,22,23]. Subjective support measures the perceived connection that an individual feels and their personal experience of being understood or supported. Objective support refers to the level of actual help they receive from society. The utilization of social support refers to the behavior an individual adopts when searching for social support. The total SSRS score is the sum of the scores of the three items and ranges from 12 to 66. Social support scores are defined as either low (≤44) or high (>44) [19].

### 2.6. Statistical Analysis

The Shapiro–Wilk test was used to test the normality of the variables. Categorical variables in the LINE and control groups were compared using the chi-square test, whereas continuous variables were compared using an independent *t*-test or repeated-measures ANOVA. The assumption of sphericity for the repeated-measures ANOVA was assessed using Mauchly’s test of sphericity, and the assumption of sphericity was met. Statistical significance was defined as a *p*-value < 0.05. Statistical analyses were performed using SAS 9.4 of the SAS System for Windows (SAS Institute Inc., SAS Campus Drive, Cary, NC, USA).

## 3. Results

**Figure 1:**
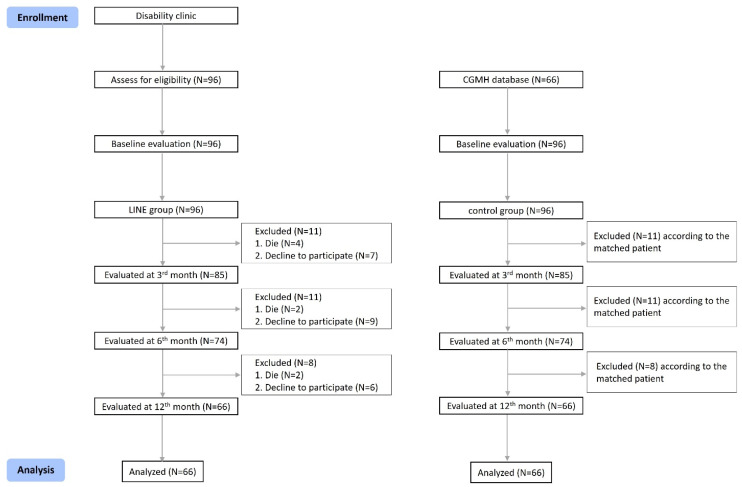
Flowchart of patient numbers in the LINE and control groups.

In total, 96 patients with disability certifications were included in the LINE group. Of these, twenty-two patients dropped out of this study because they were unable to participate due to poor cognition or communication, and eight patients died during follow-up (Figure 1). After these exclusions, 66 patients remained in the LINE group. Of these, 28 were classified as having a neuromuscular or movement-related disability and 26 as having a disability related to a mental function or the structure of the nervous system, and all the patients were taken care of by a Taiwanese caregiver. The 66 patients in the control group were matched to the participants based on age, sex, and education level and chosen from the database of patients with a disability, which was gathered from the same medical center. Thus, the demographics of both groups showed no significant differences. The results are summarized in Table 1.

**Figure 2:**
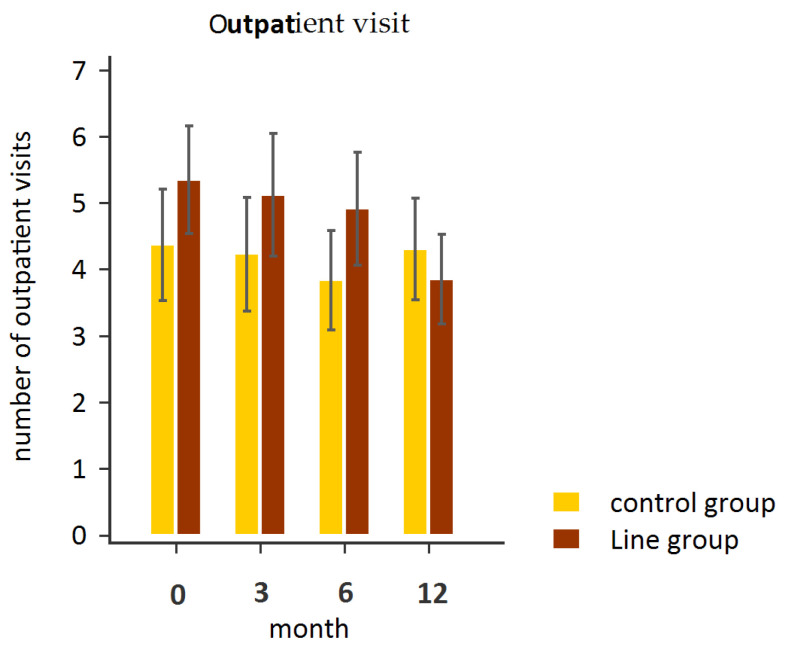
Repeated measures of outpatient visits in the LINE and control groups.

**Figure 3:**
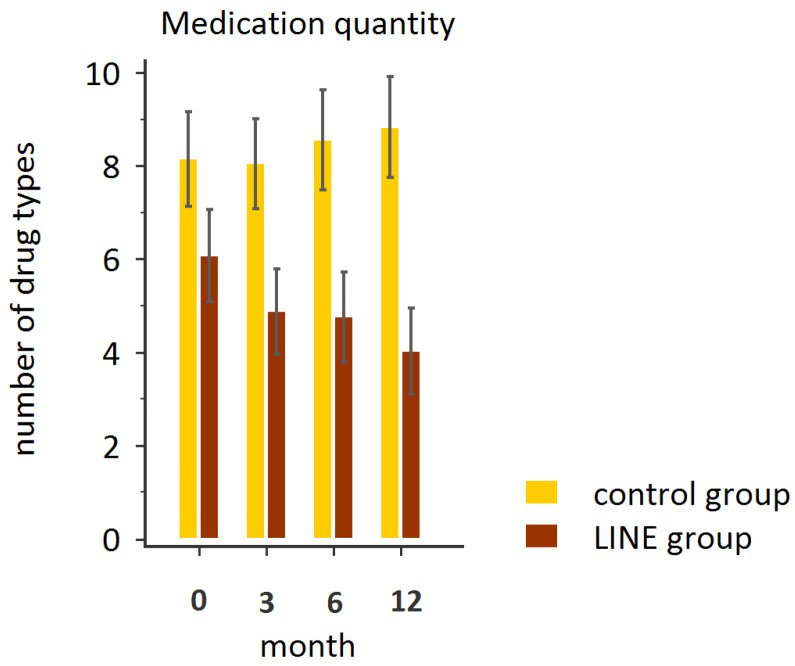
Repeated measures of the number of medications in the LINE and control groups.

After 12 months of follow-up, the patients in the LINE group demonstrated a significant reduction in outpatient clinic visits (*p* = 0.001) and the amount of medication taken (*p* < 0.001), whereas no such reductions were observed in the control group (Table 2, Figure 2 and Figure 3). These findings suggest that LINE-based integrated healthcare may have facilitated more efficient care coordination, potentially reducing the need for in-person visits and eliminating redundant prescriptions from different specialties, thereby streamlining patients’ medication use. This suggests that their access to care was improved through early interventions and timely support via instant messaging.

A repeated-measures ANOVA revealed a large effect size for the between-group difference in medication quantity (η^2^ = 0.200) and moderate effects in hospitalizations (η^2^ = 0.073). Interaction effects were small to moderate for medication quantity and outpatient visits, while effects for time alone were generally small across outcomes (Table 3), suggesting that group differences were mainly driven by the effect of the intervention rather than time alone.

A significant reduction in caregiver burden was noted in the LINE group at the 12-month follow-up. This indicates that the intervention may have provided meaningful relief for caregivers, possibly through improved communication, reduced uncertainty, and better coordination of care, but there was no significant change in WHOQOL-BREF and SSRS scores from baseline to follow-up (Table 4). This may suggest that while the intervention improved medical-related support, broader improvements in overall quality of life and perceived social support were not observed, potentially due to the multidimensional and complex nature of these outcomes.

## 4. Discussion

This database-matched controlled trial aimed to examine the effects of using instant messaging as a tool to provide integrated healthcare services. The LINE service developed in this study focused on real-time information sharing, including prompt responses to questions, the delivery of health education information, and assistance with making clinic appointments. The results of our study showed a significant reduction in the number of medications taken and clinic visits made when patients with disabilities used an integrated healthcare service provided via instant messaging. However, the WHOQOL score showed no significant differences between the two groups. In terms of the major caregivers of patients with disabilities, caregiver burden evaluation showed a gradual reduction in the burden score.

Polypharmacy, although defined in various different ways [24,25,26,27], is common among patients with a disability due to their status underlings [28]. The negative impact of polypharmacy may increase their risk of drowsiness, falls, incontinence, and Parkinson’s syndrome, leading to impaired activities of daily living (ADL) and quality of life (QOL) [26,29,30]. Polypharmacy is associated with dysphagia and malnutrition in post-stroke patients with sarcopenia [31]. Furthermore, other studies found that polypharmacy was present in long-term care patients, patients with intellectual disabilities, and older individuals [32,33]. Our findings showed that after joining the integrated healthcare service for patients with disabilities via instant messaging, patients received reduced doses of medication and made fewer visits to the clinic. This is of clinical value because it suggests a reduced risk of polypharmacy or even a reduction in unnecessary polypharmacy between different specialties. It provides a deprescribing intervention that ensures the patient’s condition remains stable and prevents patients from having to make unnecessary journeys between hospitals and their homes, saving time and decreasing the burden of transportation.

After following up with these patients for 12 months, the number of hospitalizations they experienced showed no significant difference from those recorded at baseline. This may be due to control of their underlying disease being managed through regular clinic visits and medication prescription, as well as proper care, early risk detection, and exhaustive health education. Health education is believed to be related to hospitalization frequency as it provides a person with knowledge of their disease and also encourages behavioral change [34,35], another factor covered by integrated healthcare.

Regarding QOL, our study found no significant improvement in the WHOQOL scores in the LINE group. The WHOQOL score indicates a person’s overall quality of life and general health, which are evaluated in terms of physical health, psychological health, social relations, and environmental health. In our patients, a physical impairment had resulted in a disability and handicaps, limiting their activity and resulting in their restricted participation in society. As the benefits of the integrated healthcare service for patients with disabilities via instant messaging provided mainly medical-related support, its effect on psychological, social, and environmental factors was relatively limited. Moreover, while instant messaging may provide a certain degree of emotional support, patients and their caregivers may have greater needs that are related to practical assistance in caregiving, such as respite services for the caregiver or social support from appropriate sources [36]. These forms of support are difficult to deliver through digital means alone. As a result, instant messaging-based interventions may be limited in their ability to address the complex and multidimensional aspects of QOL, particularly in domains requiring in-person assistance or broader community engagement. This may account for our finding that there was no significant improvement in QOL after receiving integrated healthcare, as QOL involves too many domains. A similar explanation also applies to the SSRS scores, in which no significant improvement was found. SSRS scores evaluate objective and subjective support and the utilization of support, which are related to interpersonal relationships with individuals, family, and society. Therefore, as the assistance provided by integrated healthcare services via instant messaging is mainly medical support and not face to face; it may not sufficiently enhance social support networks or reduce social isolation, especially among people with disabilities, who may already face challenges in community engagement. Our findings align with prior studies in suggesting that instant messaging can improve healthcare access and coordination, particularly for the management of chronic diseases [13,14]. However, unlike some reports that showed improvements in quality of life with mobile health tools [15], our study did not observe significant changes in this area, likely due to the limited ability of digital communication to address the complex physical and psychosocial needs of people with disabilities.

### Study Limitations

Our study had some limitations. First, the sample size was relatively small, and all participants were recruited from a single medical center in Taiwan. This may result in selection bias and limit the generalizability of our findings. Second, the patients had heterogeneous types of disabilities. Each disability presents different challenges; however, due to physical impairments and personal reasons, patient recruitment across this group is difficult. Also, the use of LINE for instant messaging may reflect cultural practices that are specific to Taiwan, and this may not be applicable to or feasible in other cultural or healthcare settings. Fourth, this study was a case–control study, which may have introduced confounding factors. Finally, a long-term follow-up was not performed. Further large-scale, long-term follow-up studies are warranted to assess the status of patients with disabilities who are accessing integrated healthcare services.

Some studies have illustrated the inequities in physical access to healthcare for patients with disabilities and identified their unmet healthcare needs [37,38,39,40,41,42]. However, a limited number of studies have been designed to evaluate the benefits granted by modifying aspects of healthcare provider systems and changing the methods of communication used between healthcare providers and patients with disabilities. This is the first study on incorporating instant messaging into integrated healthcare services for patients with disabilities, and it revealed that there were medical benefits and improved access to healthcare resources for patients, as well as mild relief of burden for caregivers. As disabilities restrict people’s participation in society, efforts should be made to remove and overcome these barriers and provide aid for patients with disabilities.

## 5. Conclusions

Reduced numbers of clinic visits, stable underlying disease, and the integration of medication regimens were the most beneficial aspects for patients with disabilities who participated in an integrated healthcare service delivered via instant messaging. This suggests that prompt bidirectional communication improves patients with disabilities access to healthcare systems and helps them to receive better medical care. Instant messaging can support the coordination of care and reduce the burden of clinic visits, particularly for patients with mobility or cognitive impairments. Future studies should assess long-term outcomes and explore integrating digital and in-person services to enhance the support given.

## Figures and Tables

**Table 1:** Demographic characteristics of patients in the LINE and control groups.

		LINE Group (n = 66)	Control Group (n = 66)	*p*
Age (mean ± SD)		64.1 ± 14.7	68.7 ± 13.8	0.066
Sex (n)	male	30	30	1.0
	female	36	36	
Education (n)	low literacy	14	11	0.672
	less than junior high school	17	21	
	high school or above	35	34	

**Table 2:** Between- and within-group comparisons of the LINE and control groups.

	LINE Group				*p*	Control Group				*p*	*p* Between Groups		
	Baseline	3 Months	6 Months	12 Months	Within Group	Baseline	3 Months	6 Months	12 Months	Within Group	*p*1	***p*** **2**	***p*** **3**
outpatient visit (mean ± SD; median)	5.4 ± 3.3 5	5.1 ± 3.7 4	4.9 ± 3.5 4	3.9 ± 2.7 3	0.001 *	4.4 ± 3.4 3	4.2 ± 3.5 3	3.8 ± 3.0 3	4.3 ± 3.1 3	0.125	0.415	0.022 *	<0.001 *
hospitalization (mean ± SD; median)	0.1 ± 0.3 0	0.1 ± 0.4 0	0.1 ± 0.3 0	0.2 ± 0.6 0	0.505	0.1 ± 0.3 0	0.0 ± 0.1 0	0.0 ± 0.2 0	0.0 ± 0.1 0	0.865	0.346	0.396	0.764
medication quantity (mean ± SD; median)	6.9 ± 4.0 6	4.9 ± 3.7 4	4.8 ± 3.9 5	4.0 ± 3.7 3.5	<0.001 *	8.1 ± 4.1 8	8.1 ± 3.9 7.5	8.6 ± 4.3 8	8.8 ± 4.4 8.5	0.063	0.028 *	0.165	0.026 *

* *p* represents within-group comparison; *p*1, *p*2, and *p*3 represent comparisons between the control and LINE groups at 3 months, 6 months, and 12 months, respectively. * *p*-value < 0.05.

**Table 3:** Partial eta-squared (η^2^) values from repeated-measures ANOVA comparing the LINE and control groups.

Outcome	Group Effect (η^2^)	Time Effect (η^2^)	Interaction Effect (η^2^)
Outpatient visits	0.012	0.036	0.040
Hospitalization	0.073	0.0019	0.006
Medication quantity	0.200	0.018	0.057

**Table 4:** Social support, quality of life, and caregiver burden evaluations in the LINE group.

	Baseline	3 Months	6 Months	12 Months	*p*
SSRS (mean ± SD)	57.5 ± 10.0	57.8 ± 9.9	57.5 ± 10.7	56.1 ± 10.5	0.205
WHOQOL-BREF (mean ± SD)	49.8 ± 6.3	48.8 ± 6.3	50.8 ± 9.1	49.2 ± 7.0	0.588
CBI (mean ± SD)	45.7 ± 15.6	43.9 ± 14.6	42.5 ± 16.0	41.8 ± 16.4	0.024 *

* *p*-value < 0.05. Abbreviations: WHOQOL-BREF (The World Health Organization Quality of Life Brief Version); SSRS: social support rating scale; CBI: Caregiver Burden Inventory.

## Data Availability

The original contributions presented in this study are included in the article. Further inquiries can be directed to the corresponding author.

## Abbreviations

The following abbreviations are used in this manuscript:

CBI	Care burden index
WHOQOL-BREF	The World Health Organization Quality of Life Brief Version
SSRS	Social support rating scale
ADL	Activities of daily living
QOL	Quality of life
